# Follicle Rescue From Prepubertal Ovaries After Recent Treatment With Cyclophosphamide—An Experimental Culture System Using Mice to Achieve Mature Oocytes for Fertility Preservation

**DOI:** 10.3389/fonc.2021.682470

**Published:** 2021-09-24

**Authors:** Xia Hao, Amandine Anastácio, Laia Viñals-Ribé, Ana Santamaria Lacuesta, Christina Diakaki, Sara Alonso de Mena, Kui Liu, Kenny A. Rodriguez-Wallberg

**Affiliations:** ^1^ Department of Oncology and Pathology, Karolinska Institutet, Stockholm, Sweden; ^2^ Laboratory of Translational Fertility Preservation, BioClinicum, Stockholm, Sweden; ^3^ Shenzhen Key Laboratory of Fertility Regulation, Center of Assisted Reproduction and Embryology, The University of Hong Kong-Shenzhen Hospital, Shenzhen, China; ^4^ Department of Obstetrics and Gynecology, Li Ka Shing Faculty of Medicine, The University of Hong Kong, Hong Kong, Hong Kong, SAR, China; ^5^ Department of Reproductive Medicine, Division of Gynecology and Reproduction, Karolinska University Hospital, Stockholm, Sweden

**Keywords:** female fertility preservation, cyclophosphamide, *in vitro* culture and maturation, prepubertal ovary, chemotherapy, ovarian follicle isolation

## Abstract

Ovarian tissue cryopreservation is the only feasible method for fertility preservation in prepubertal girls that will undergo gonadotoxic chemotherapy. To date, the only clinical use of cryopreserved tissue is by a later tissue retransplantation to the patient. Clinical challenges in fertility preservation of very young patients with cancer include time constraints that do not allow to retrieve the tissue for cryopreservation before starting chemotherapy and the preclusion of future ovarian tissue transplantation due to the risk of reintroduction of malignant cells in patients with systemic diseases. To overcome these two challenges, we investigated using an experimental model the feasibility of retrieving secondary follicles from ovaries of prepubertal mice after cyclophosphamide (CPA) treatment in increasing doses of 50, 75, and 100 mg/kg. The follicles were thereafter cultured and matured *in vitro*. The main outcomes included the efficiency of the method in terms of obtained matured oocytes and the safety of these potentially fertility preservative procedures in terms of analyses of oocyte competence regarding normality of the spindle and chromosome configurations. Our findings demonstrated that it was feasible to isolate and culture secondary follicles and to obtain mature oocytes from prepubertal mice ovaries recently treated with CPA. The efficiency of this method was highly demonstrated in the 100 mg/kg CPA group, with near 90% follicle survival rate after 12 days’ culture, similarly to control. Around 80% of the follicles met the criteria to put into maturation, and more than 40% of them achieved metaphase II, with normal spindle and chromosome configurations observed. Suboptimal results were obtained in the 50 and 75 mg/kg CPA groups. These paradoxical findings towards CPA dose might probably reflect a more difficult selection of damaged growing follicles from ovaries recently treated with lower doses of CPA and a hampered ability to identify and discard those with reduced viability for the culture.

## Introduction

Improvements in diagnostic methods allowing early cancer diagnosis and improvements in cancer treatment have both increased cancer patients’ survival rate globally. However, cancer treatment, especially chemotherapy and radiotherapy, can cause premature ovarian failure and infertility (1). For a young cancer survivor, this is a hard pathway to face. Thus, efforts have been made to develop methods for fertility preservation, and current guidelines recommend timely discussions with young adult patients, children, and their families on feasible fertility preservative methods as early as possible before the treatment starts aiming at offering the full range of options (2, 3).

Although well-established female fertility preservative methods including the cryopreservation of mature oocytes or embryos are available worldwide for adult patients, these are not applicable to young girls. Ovarian tissue cryopreservation can be offered instead in these cases (4). The method does not require hormonal stimulation, and the tissue is usually retrieved using minimally invasive surgery, which has been reported by programs for fertility preservation (5). However, many young patients need to start a treatment without delay, and data are lacking regarding the usefulness of ovarian tissue retrieved after the chemotherapy rounds have been already initiated.

Up to date, the only currently developed method to regain fertility using the cryopreserved ovarian tissue is by retransplantation of the tissue to the patient. The ovarian follicles will then grow and develop to allow the performance of assisted reproductive treatments or even natural conceptions (6). A few hundreds of successful cases using cryopreserved ovarian tissue have been reported worldwide; however, a high number of women cannot undergo transplantation due to the risk of reseeding malignant cells back within the transplanted tissue. There is hence an urgent need for developing novel methods to use the ovarian tissue in the future, as the numbers of women undergoing these procedures are increasing (7).

In this study, we approached two challenging clinical situations using a translational research model. We wished to investigate the feasibility of follicle isolation from prepubertal mice ovaries recently treated with a gonadotoxic cytostatic drug, cyclophosphamide (CPA) (8), and the reproductive potential of these follicles regarding the final achievement of mature competent oocytes. Three different doses of CPA were tested *vs*. a control group without treatment. In all groups, isolated follicles were thereafter *in vitro* cultured and matured. The culture system allowed the evaluation of individual follicle growth, hormonal production, oocyte maturation, and spindle structure and chromosomal configurations in mature oocytes. To our knowledge, there is limited knowledge on the feasibility of performing fertility preservative procedures after gonadotoxic cancer treatment has been initiated, especially at prepubertal stage (9, 10).

## Materials and Methods

All chemicals used in this study were purchased from Sigma-Aldrich^®^ or Gibco, Thermo Fisher Scientific^®^, unless otherwise indicated.

### Animals and Grouping

Twenty in-house breeding 12-day-old B6CBA/F1 female mice were randomly assigned (n = 5/group) into three groups treated with different doses of CPA (100, 75, or 50 mg/kg) or into a control group without treatment. CPA was freshly prepared in a 0.9% NaCl solution and intraperitoneally injected. All the mice were sacrificed 3 days after CPA injection, and the ovaries were collected into Leibovitz’s 15 media supplemented with 10% fetal bovine serum, 100 IU/ml penicillin, and 100 µg/ml streptomycin, at 37°C. The doses of CPA chosen for this study have been validated in previous experimental studies that have also considered their equivalence to human treatment (11, 12).

All animal procedures were approved by Karolinska Institutet and the regional ethics committee for animal research in accordance with the Animal Protection Law, the Animal Protection Regulation, the Regulation of the Swedish National Board for Laboratory Animals, identified Dnr 1372 (date: January 24, 2018). All procedures were conducted in accordance with accepted standards of humane animal care.

### Follicle Isolation, *In Vitro* Culture, and Maturation

Follicle isolation was performed immediately after ovary collection. Under a stereomicroscope (Nikon^®^), the ovaries were cleaned up of the surrounding tissue, and follicles were isolated mechanically using micro-fine U-100 insulin syringes (0.3 ml, BD Medical). Secondary follicles with 100–130 µm diameter, two or more layers of granulosa cells, a round and central oocyte, and some theca cells attached were selected for culture. Ovarian follicle culture was performed as previously described (10, 13, 14). Briefly, selected secondary follicles were individually cultured in drops of a droplet system (20 µl per drop, 10 drops per dish) containing culture medium. The culture medium was α-minimal essential medium (α-MEM) GlutaMAX supplemented with 5% fetal bovine serum, 10 µg/ml transferrin, 5 µg/ml insulin, and 100 mIU/ml recombinant follicle-stimulating hormone (GONAL-F). The droplet system was covered with mineral oil and kept in a humidified incubator at 37°C with 5% CO_2_. Isolation, selection, and start of culture day was designated as day 0 and the last day as day 12. Every other day, the follicles were observed under an inverted microscope (100×) (Nikon^®^) to record morphological characteristics and follicular size using a calibrated ocular micrometer (Nikon^®^). Follicular size was estimated as the mean diameter obtained with two perpendicular measures including the granulosa cell mass without the theca cells. Culture medium was renewed every other day by collecting 10 µl of culture medium and adding 10 µl of fresh culture medium. On culture days 4, 8, and 12, the culture medium collected was diluted in 90 µl α-MEM GlutaMAX with bovine serum albumin (40 mg/ml) and stored at −20°C for further analysis.

On day 12 of culture, follicles that reached at least 200 µm of diameter, presented a clear granulosa cell proliferation, and had a visible round oocyte were classified as growing follicles that survived throughout the *in vitro* culture. Additionally, follicles with at least 450 µm diameter were selected for *in vitro* maturation. *In vitro* maturation was performed by transferring the selected follicles at day 12 to a microdrop system similar to the culture system, but in which 1.5 IU/ml recombinant human chorionic gonadotrophins and 5 ng/ml recombinant epidermal growth factor were added to the culture medium. Follicles were incubated in a humidified atmosphere at 37°C and 5% CO_2_, and maturation status was verified 16–20 h later. After verifying the cumulus–oocyte complex formation, the oocytes of the follicles that formed cumulus–oocyte complex were denuded, and their maturation status were evaluated under an inverted microscope. Oocytes with a visible polar body were classified as mature oocytes (metaphase II, MII).

### Hormone Assays

The culture medium collected on days 4, 8, and 12 were used to measure the secretion of 17β-estradiol and anti-Müllerian hormone (AMH). Hormonal assays were performed using commercially available enzyme-linked immunoassay kits for 17β-estradiol (ab108667, Abcam) and AMH (RK02588, ABclonal) following the manufacturers’ protocols. The limits of sensitivity for 17β-estradiol and AMH were 8.68 and 53.3 pg/ml, respectively. For each hormone, duplicate measurements were performed using the collected culture medium at days 4, 8, and 12 of culture. For each time, in each group, the culture media of five follicles with similar growth features and from which resulted mature oocytes were pooled together to reach the required volume sample amount for the assay.

### Spindle and Chromosome Analysis of *In Vitro* Matured Oocytes

After *in vitro* maturation (IVM), denuded mature oocytes (MII) were selected and washed in a washing buffer (Dulbecco’s phosphate-buffered saline) containing 0.1% polyvinyl alcohol. The oocytes were then fixed with 2% formaldehyde in washing buffer containing 0.2% Triton X-100 for 40 min. After fixation, the oocytes were incubated overnight at 4°C in a blocking buffer (washing buffer supplemented with 1% bovine serum albumin). Then, the oocytes were incubated for 40 min in the blocking buffer supplemented with 10% fetal bovine serum. Antibodies used were diluted in the blocking buffer. The oocytes were incubated with mouse monoclonal anti-α-tubulin antibody (T9026, 1:1,000) for 45 min followed by a 40-min incubation with Alexa Fluor 488-labeled goat antimouse IgG antibodies (ab150113, Abcam, 1:200) at 37°C. Then, 10 μg/ml propidium iodide (81845) was added to the oocytes for 20-min incubation. Finally, oocytes were mounted between a coverslip and a microscope slide with Prolong Diamond Antifade mountant (P36965, Invitrogen). All the above-described procedures were performed under a stereomicroscope. The slides were kept at 4°C until confocal imaging.

Labeled tubulin and chromatin were assessed using a Nikon Eclipse Ti microscope equipped with the appropriate filter sets for analyzing Alexa Fluor 488 and propidium iodide with 100× oil immersion objective. Four to six MII oocyte images in each group were captured with an Andor iXon Ultra camera and analyzed using NIS Elements program. Image analyses were performed using Fiji software.

### Statistical Analysis

Statistical analyses were performed using the GraphPad Prism 8.4.3 software package. Differences in follicular sizes, hormones levels on different culture days, and follicle yield per ovary between groups were tested by multiple t-tests. Comparisons of parameters other than follicle yield in **Table 1** between CPA treated groups and control were performed by chi-square test (two-sided).

**Table 1:** Follicle yields per ovary, *in vitro* growth characteristics, and oocyte maturation status achieved by CPA-treated groups in comparison with controls.

	Ovaries	Cultured follicles	Follicle yields/ovary	Attached within first 2 days	Survival (Day 12)	Put into maturation	MII oocytes
	N	N	Mean ± SD	N (% = 100%*N/Cultured follicles)			
Control	9	134	14.9 ± 4.4	113 (84.3%)	119 (88.8%)	107 (79.8%)	69 (51.5%)
50 mg/kg	9	170	18.9 ± 6.4	145 (85.3%)	133 (78.2%)^a^	115 (67.6%)^a^	56 (32.9%)^b^
75 mg/kg	9	211	23.6 ± 5.5^b^	178 (84.4%)	172 (81.5%)	148 (70.1%)^a^	61 (28.9%)^c^
100 mg/kg	9	144	16.0 ± 5.1	130 (90.3%)	129 (89.6%)	110 (76.4%)	60 (41.7%)

^a^p < 0.05; ^b^p < 0.01; ^c^p < 0.001

## Results

### Secondary Follicle Isolation Yield, *In Vitro* Growth, and Maturation

A total of nine ovaries per group were used in this study. The yield of secondary follicles isolated per ovary did not differ significantly between 50 or 100 mg/kg CPA groups *vs*. control; however, in the 75 mg/kg group, the yield was significantly higher *vs*. control (*p* < 0.01, **Table 1**).

**Figure 1:**
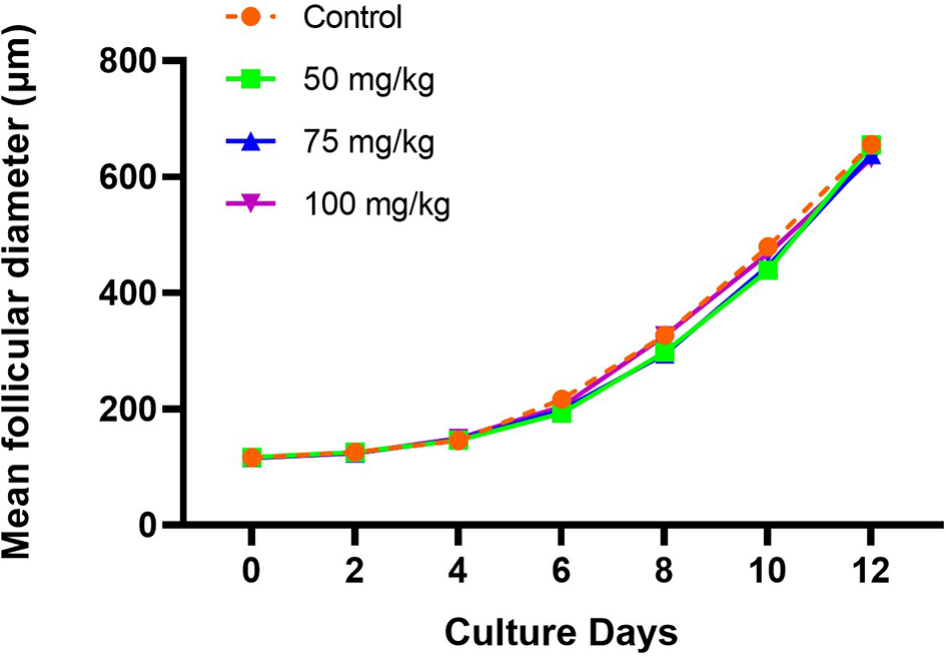
Follicular growth curve in each group during the culture (X, culture days; Y, mean follicular diameter, µm).

The *in vitro* growth behavior of follicles during culture was similar among all groups regarding percentages of follicles attached to the culture dish during the first 2 days (**Table 1**) and growth curve features (**Figure 1**). Overall, the mean follicular size steadily increased throughout the culture but with different paces at different culture periods, with slower growth during the first half of culture and faster during the second half (**Figure 1**). The initial mean follicle diameter was 116.2 µm among all the groups (**Table 2**). By the end of culture, follicles in all groups reached mean sizes ranging from 631.8 to 655.9 µm (**Table 2**) without significant differences. After 12 days in culture, nearly 90% of the follicles from the control and the 100 mg/kg groups survived. However, a lower follicle survival was observed in the 50 mg/kg CPA group (78.2%) *vs*. control (88.8%), *p* < 0.05 (**Table 1**). By the end of culture, significantly lower percentages of follicles reached the criteria to put into maturation in the 50 or 75 mg/kg CPA groups *vs*. control, whereas similar percentages were observed in the 100 mg/kg and control groups.

**Table 2:** Dynamic follicular sizes in each group during the culture (mean follicle diameter ± standard deviation, µm).

	D0	D2	D4	D6	D8	D10	D12
Control	116.0 ± 9.3	125.5 ± 14.6	145.8 ± 27.3	217.3 ± 102.8	326.8 ± 166.8	479.6 ± 191.9	655.9 ± 190.0
50 mg/kg	116.9 ± 8.6	125.5 ± 10.8	146.6 ± 27.3	192.7 ± 83.9	298.1 ± 157.6	439.1 ± 209.6	655.0 ± 199.3
75 mg/kg	116.4 ± 8.3	124.4 ± 12.4	147.5 ± 27.1	198.4 ± 73.1	294.7 ± 144.6	446.2 ± 187.0	638.0 ± 193.5
100 mg/kg	115.6 ± 8.5	124.5 ± 12.8	149.5 ± 32.0	204.3 ± 92.5	326.3 ± 169.9	467.6 ± 192.2	631.8 ± 179.3

After IVM, more mature oocytes were obtained in the control group (51.5%) compared with the 50 and 75 mg/kg groups, where 32.9% and 28.9% were matured, respectively (**Table 1**). In the group treated with 100 mg/kg CPA, a similar percentage of mature oocytes was obtained compared to the control.

### Hormone Assays

**Figure 2:**
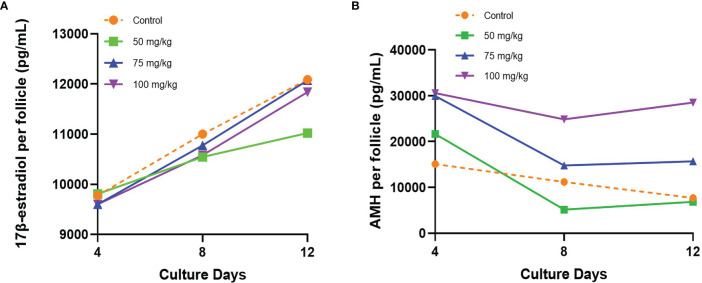
**(A)** 17β-estradiol and **(B)** AMH secreted by individual follicle into the culture medium during the culture.

Follicle secretion of 17β-estradiol determined in culture media was similar among all groups, increasing from day 4 to 12 (**Figure 2A**). On the last day of culture (day 12), the levels of 17β-estradiol detected were similar among controls and the 75 and 100 mg/kg CPA groups. However, a lower secretion of 17β-estradiol was detected in the 50 mg/kg CPA group, compared to control (11,023.57 pg/ml *vs*. 12,087.9 pg/ml, *p* < 0.005).

Initial AMH secretion measured in culture medium on day 4 showed similar levels between control and 50 mg/kg CPA groups, whereas a significantly higher initial AMH secretion was found in the 75 and 100 mg/kg CPA groups *vs*. control (**Figure 2B**). Over time, the AMH secretion gradually declined in the 50 and 75 mg/kg CPA groups and control, and the levels at day 12 did not differ among those groups *vs* control. However, the 100 mg/kg CPA group maintained a steady pace throughout the culture, and on day 12, the AMH level was 3.7 times of control (*p* < 0.0001).

### Spindle and Chromosome Configurations in Mature Oocytes

**Figure 3:**
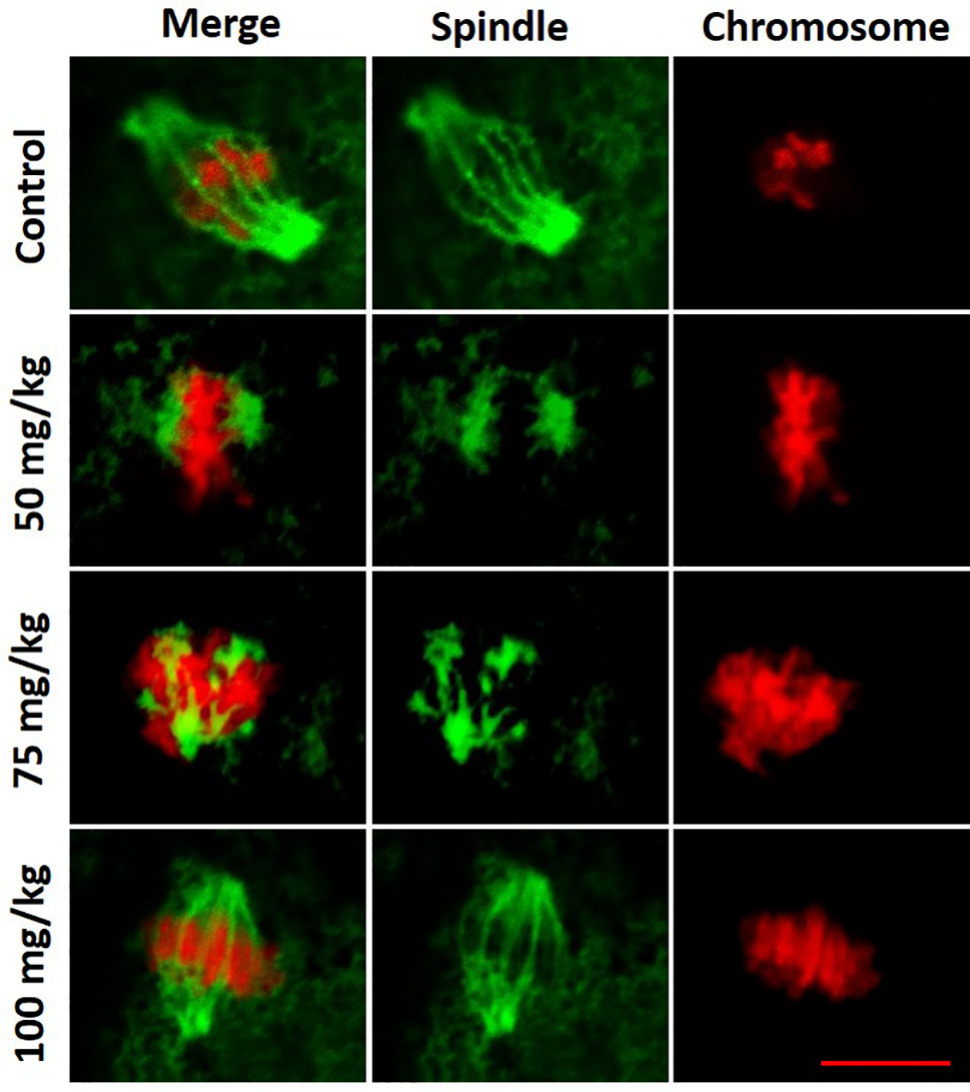
Representative figures of spindle and chromosome configurations in MII oocytes from each group. Spindle fibers were detected by immunofluorescence for α-tubulin (green), while DNA was stained with propidium iodide (red). Scale bar, 10 µm.

In a normal MII oocyte, the spindle is bipolar barrel shaped, and the chromosomes are well-aligned on the metaphase equator in the center of the spindle. Within the MII oocytes observed, there was a trend that the spindle and chromosome structures in 100 mg/kg CPA group were more similar to control, whereas spindle and chromosome abnormalities were frequently observed in 75 and 50 mg/kg CPA groups. As shown in **Figure 3**, mature oocytes in the control group displayed organized chromosomes at the center of the bipolar barrel-shaped spindle. Whereas in the 75 and 50 mg/kg CPA groups, chromosome misalignment and spindle defects were observed in mature oocytes. Chromosomes were, even though at the center of the spindle, not well-aligned on the metaphase equator; meanwhile fragmentary spindles were also observed. In mature oocytes obtained from the 100 mg/kg CPA group, the spindle was bipolar barrel shaped, and the chromosomes were well-aligned on the metaphase equator.

## Discussion

This study was designed to investigate the feasibility of obtaining mature oocytes after *in vitro* culture of follicles isolated from prepubertal mice recently treated with CPA. The follicle culture and oocyte maturation methods have been previously validated in mice, and pups have been obtained using those methods (13–15). Spindle and chromosome arrangements were analyzed in this study to evaluate the quality of the mature oocytes obtained.

The ovary is a complex organ, where multiple regulations among activated follicles determine the ultimate fate of each individual follicle, whether it will proceed towards further growth or become atretic, resulting in the elimination of more than 90% of the activated follicles (16). Meanwhile, there is a critical surveillance system in the ovary, to remove dead follicles and follicles with damage (17–19). In our study, ovaries post CPA treatment may have had a proportion of secondary follicles physiologically impaired, and some were probably already targeted by the surveillance system due to CPA-induced damage. In our experimental conditions, we removed the secondary follicles from the ovarian environment to culture them individually, allowing them to escape the physiological inhibition and the surveillance system with the final aim to grow till mature oocytes.

Our findings showed that a large proportion of secondary follicles isolated from the ovaries of prepubertal mice after recent CPA treatment were not seriously affected by the CPA treatment. Those follicles could be supported to grow, and had spindle and chromosome configurations that were normal in the mature oocytes obtained after IVM, supporting the feasibility of our proposed method. Meanwhile, we found an interesting phenomenon towards the relationship between the efficiency of this method and the doses of CPA used, and we speculate this is somehow related to the mechanisms of how CPA induces primordial follicle depletion (PFD) in the ovaries.

According to the follicle selection criteria used in this culture system (13, 14), the yield of follicles/ovary obtained in the 75 mg/kg group was significantly higher and in the 50 mg/kg group slightly higher than that of control, whereas follicle yield was smaller in the 100 mg/kg group. This could support an early overactivation mechanism, which has been proposed to explain the final PFD induced by gonadotoxic chemotherapy (20–23), or it could indicate that in the two lower-dose groups of this study, the CPA dose was not high enough to immediately damage the follicles or trigger apoptosis in them. In 50 and 75 mg/kg groups increased activation might have allowed to pick more secondary follicles for culture; however, at 100 mg/kg, the follicle yield could have been additionally affected because the activated follicles were more rapidly damaged due to a high CPA dose, and many of them did not met the criteria to be cultured.

The gonadal toxicity of CPA is dose dependent, as previously demonstrated (24, 25). In our study, the observed similar culture outcomes between 100 mg/kg CPA and the control groups, while poorer outcomes in 50 and 75 mg/kg CPA groups, were surprising. These might indicate that the initial selection of potentially viable secondary follicles was easier in the 100 mg/kg group, due to more serious recent CPA treatment induced damage causing morphologically recognizable changes. The visible changes allowed to discard follicles with damage and to select follicles that had survived the damage and probably were qualified to overcome the surveillance system, while this selection was more difficult in the 50 and 75 mg/kg groups. Thus, a higher proportion of secondary follicles with minor but not morphologically detectable damage was selected for culture in the 50 and 75 mg/kg groups. However, during culture, it was evident that the competency of these follicles was impaired, and a lower survival rate was observed after 12 days of *in vitro* culture. Additionally, lower percentages of cultured follicles from these groups reached the criteria for IVM, lower percentages of MII oocytes were obtained, and abnormal spindle and damaged chromosome structures were more frequently observed in the oocytes of these two groups.

The observed higher levels of AMH in 75 and 100 mg/kg CPA groups on day 4, and continuously higher in 100 mg/kg CPA group till day 12, might also support plausible primordial follicle activation after CPA *in vivo* treatment (20–23). This is not contradictory to the observation of reduced serum AMH levels by CPA treatment in studies *in vivo* (26, 27). Overactivation *in vivo* can initially lead to follicles sense through molecular communications and induce enhanced secretion of AMH to inhibit further overactivation through a paracrine regulation (28–30). Thereafter, the activated follicles may suffer direct damage by CPA or by lacking of growth support; thus, the AMH secretion becomes reduced in the long run. In this study, 72 h after CPA treatment, the activated follicles were isolated and individually cultured *in vitro*, leaving them free from subsequent CPA damage, but the initial effect that enhanced AMH production seemed to be kept, and the length of duration keeping this feature seemed to be dose dependent. As an alternative explanation, AMH could promote the growth of preantral follicles during *in vitro* culture through an autocrine effect, as supported by some studies (31–33). In our study, follicles isolated from 100 mg/kg CPA-treated mice ovaries might had been real survivors, secreting more AMH to promote their own growths. More investigations will be needed to further explain this phenomenon.

Since it is known that CPA is genotoxic (34), thus it is important to investigate the normality of the mature oocytes obtained after recent CPA treatment to guarantee the safety of our method. In addition to spindle and chromosome structure analysis, more investigations should be performed in the future, such as chromosome aberration tests and the final fertilization ability of the oocytes to evaluate normality of embryo development. On the other hand, clinical data from young female patients that have undergone ovarian tissue cryopreservation after several chemotherapy rounds and even after hematologic stem cells transplantation (HSCT) has demonstrated presence of primordial ovarian follicles in the cryopreserved tissue when the ovarian tissue was retrieved during childhood or adolescence (35). Moreover, ovarian tissue retrieved following HSCT conditioning at pubertal age has demonstrated full functionality after retransplantation, allowing two normal pregnancies, as recently reported (36).

Our experimental model deserves further translational investigation using human ovarian tissue. Methods for isolation and culture of human ovarian follicles obtaining mature oocytes have been reported (37). Further development of the methods hereby described is needed for the establishment of fertility preservative methods that can be performed after initiation of gonadotoxic chemotherapeutic treatment and that overcome the need of ovarian tissue retransplantation.

## Data Availability Statement

The raw data supporting the conclusions of this article will be made available by the authors, without undue reservation.

## Ethics Statement

The animal study was reviewed and approved by Karolinska Institutet and the regional ethics committee for animal research.

## Author Contributions

XH, AA, and KR-W designed the experimental setting for the study. XH, AA, LV-R, ASL, CD and SAdM performed the experiments. XH had the main responsibility for data analysis and wrote the first manuscript draft. All authors critically revised the manuscript. K-RW provided administrative support and funding. All authors contributed to the article and approved the submitted version.

## Funding

This study was funded by the Swedish Childhood Cancer Foundation (PR2016-0115, PR2020-0136), the Swedish Cancer Society (CAN 2017/704, 20 0170 F), the Swedish Research Council (Dnr 2020-02230), Radiumhemmets Research Funds Grant for clinical researchers 2020–2025, the Stockholm County Council (FoUI-953912), and the Karolinska Institutet Research grants in pediatrics from the Birgitta and Carl-Axel Rydbeck Donation, 2020-00339 to KR-W. Doctoral candidate XH has been supported by the China Scholarship Council.

## Conflict of Interest

The authors declare that the research was conducted in the absence of any commercial or financial relationships that could be construed as a potential conflict of interest.

## Publisher’s Note

All claims expressed in this article are solely those of the authors and do not necessarily represent those of their affiliated organizations, or those of the publisher, the editors and the reviewers. Any product that may be evaluated in this article, or claim that may be made by its manufacturer, is not guaranteed or endorsed by the publisher.

## Abbreviations

CPA, cyclophosphamide; α-MEM, α-minimal essential medium; MII, metaphase II; AMH, anti-Müllerian hormone; IVM, *in vitro* maturation; PFD, primordial follicle depletion; HSCT, hematologic stem cells transplantation.
